# The Proctologist for March, 1911*The *Proctologist* is published quarterly in St. Louis, Dr. R. H. Barnes, Editor.

**Published:** 1911-03

**Authors:** 


### The Proctologist for March, 1911.*

The *Proctologist* for March, 1911, will contain:

"Fistula," by Mr. Fred C. Wallis, F. R. C. S., Surgeon St. Mark's Hospital, London, England.

"Hypogastric Versus Iliac Colostomy," by Mr. F. Swinford Edwards, F. R. C. S., Surgeon St. Mark's Hospital, London, England.

"The Clinical Aspect of Sigmoid Diverticula," by Mr. W. Maxwell Telling, M. R. C. P. I., Leeds, England.

"Severe Ulceration of the Rectum," by Mr. H. Graeme Anderson, M. B., London, England.

"Submucous Anal Fistula," by Mr. Harrison Cripps, F. R. C. S., Surgeon St. Bartholomew's Hospital, London, England.

"Treatment of Cancer of the Rectum," by Mr. Hamilton Drummond, Newcastle-on-the-Tyne, England.

---

*The *Proctologist* is published quarterly in St. Louis, Dr. R. H. Barnes, Editor.

Can you afford to do without this journal that gathers the literature of the world, for one dollar a year, of a specialty that represents one out of seven general hospital cases?

During your college course were you given instruction in proctology?

---

The resolution adopted by the Nebraska Swine Breeders' Association at Omaha, asking the next Legislature to appropriate $7000 to equip a hog cholera serum laboratory at the Nebraska State farm, will achieve their object beyond a doubt. The farmer vote is not a negligible quantity, and when agriculturists and stock breeders demand legislation the politicians accede to it with alacrity. Physicians, hygienists, humanitarians, having in view the saving of precious human lives, often stand at the doors of legislative halls asking for appropriations or legal enactments to assist them in their unselfish endeavors, and the busy legislators heed them not. In the last analysis it is the fault of the well-wishers of society. They are not united.—*Lancet-Clinic.*

